# Novel Fucoidan Pharmaceutical Formulations and Their Potential Application in Oncology—A Review

**DOI:** 10.3390/polym15153242

**Published:** 2023-07-29

**Authors:** Nikolay Zahariev, Plamen Katsarov, Paolina Lukova, Bissera Pilicheva

**Affiliations:** 1 Department of Pharmaceutical Sciences, Faculty of Pharmacy, Medical University of Plovdiv, 15A Vassil Aprilov Blvd, 4002 Plovdiv, Bulgaria; nikolay.zahariev@mu-plovdiv.bg (N.Z.); bisera.pilicheva@mu-plovdiv.bg (B.P.); 2 Research Institute, Medical University of Plovdiv, 15A Vassil Aprilov Blvd, 4002 Plovdiv, Bulgaria; 3 Department of Pharmacognosy and Pharmaceutical Chemistry, Faculty of Pharmacy, Medical University of Plovdiv, 15A Vassil Aprilov Blvd, 4002 Plovdiv, Bulgaria; paolina.lukova@mu-plovdiv.bg

**Keywords:** polysaccharides, fucoidan, biological activities, micro- and nanoparticles, targeted drug delivery

## Abstract

Fucoidan belongs to the family of marine sulfated, L-fucose-rich polysaccharides found in the cell wall matrix of various brown algae species. In the last few years, sulfated polysaccharides have attracted the attention of researchers due to their broad biological activities such as anticoagulant, antithrombotic, antidiabetic, immunomodulatory, anticancer and antiproliferative effects. Recently the application of fucoidan in the field of pharmaceutical technology has been widely investigated. Due to its low toxicity, biocompatibility and biodegradability, fucoidan plays an important role as a drug carrier for the formulation of various drug delivery systems, especially as a biopolymer with anticancer activity, used for targeted delivery of chemotherapeutics in oncology. Furthermore, the presence of sulfate residues with negative charge in its structure enables fucoidan to form ionic complexes with oppositely charged molecules, providing relatively easy structure-forming properties in combination with other polymers. The aim of the present study was to overview essential fucoidan characteristics, related to its application in the development of pharmaceutical formulations as a single drug carrier or in combinations with other polymers. Special focus was placed on micro- and nanosized drug delivery systems with polysaccharides and their application in the field of oncology.

## 1. Introduction

Cancer is one of the major causes of death worldwide. The possible complications during therapy, which can be due to inadequate biodistribution of chemotherapeutics in the organism, should not be underestimated. Current pharmaceutical technology aims at developing drug delivery systems with improved biodistribution of the chemotherapeutics, reduced therapy-related side effects and toxicity and therefore improving the therapeutic efficacy. In recent years the rapid development of micro- and nanotechnologies has led to the formulation of innovative drug systems, which take their place in contemporary oncological therapeutic approaches. Much of the research nowadays is focused on the utilization of natural polymers such as polysaccharides, proteins and lipids as drug carriers for chemotherapeutic agents. Biopolymers are preferred materials for developing drug formulations due to their biocompatibility, biodegradability, low toxicity and low immunogenicity [1]. Natural polymers can be conjugated with specific ligands leading to the formation of pharmaceutical systems with improved functional properties and targeted drug delivery. Some biopolymers possess biological activities and, in addition to being carriers, they can also show a synergistic therapeutic effect with the incorporated active substances [2]. Marine polysaccharides, more specifically, have recently been an object of great scientific interest due to their unique structural and physicochemical properties. Fucoidan is a sulfated polysaccharide which is mainly extracted from brown seaweed. Depending on their origin and type of extraction, fucoidans have different molecular weights and degrees of sulfation, which can affect the biological effects of the polysaccharide and the characteristics of its formulations [1,2,3]. Recently the application of fucoidan in the field of pharmaceutical technology has been widely investigated. This polysaccharide plays an important role as a drug carrier for the formulation of various drug delivery systems, especially as a biopolymer with anticancer activity, used for targeted delivery of chemotherapeutics in oncology [4]. The polymer is characterized with easy structure-forming properties and can be used as a single carrier or in combination with other polymers for the formulation of innovative anticancer strategies with improved therapeutic efficacy, including micro- and nano-sized drug delivery systems. 

The present study aims at overviewing some of the essential fucoidan characteristics and biological activities, related to its application in the development of pharmaceutical formulations, with focus on the strategies used for the design of fucoidan-based micro- and nanostructures and their potential used as drug delivery systems in cancer therapy. 

## 2. Materials and Methods

The review article is based on the literature found in the databases of PubMed, Web of science and Science Direct. The performed survey was within the year interval 2000–2023, and 202 references were selected for the review. The choice of publications was made based on the relevance of the publications to the topic, the research methodology and the research results. The cited publications include systematic reviews, research articles and book chapters.

## 3. Fucoidan Sources and Chemical Structure

Fucoidan belongs to a family of sulfated, L-fucose-rich polysaccharides found in the cell wall matrix of various species of marine brown algae (*Phaeophyta*: Laminariaceae, Fucaceae, Chordariaceae and Alariaceae). Fucoidan can also be obtained from sea cucumbers (*Holothuroidea*: Stichopodidae, Holothuriidae), sea urchin eggs (*Echinoidea*: Strongylocentrotidae, Arbaciidae) and sea grasses (Cymodoceaceae) [5]. Depending on the extracted species, growth environment and extraction method, fucoidan exhibits different structure and molecular weight (Table 1) [6].

**Figure 1:**
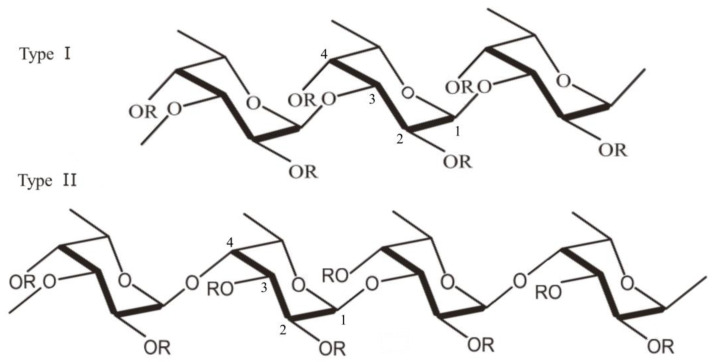
Structure of fucoidan: α-(1→3)-α-L-fucopyranose backbone linked to sulfate radicals at the C2 and C4 position (Type I) and α-(1→3)- and α-(1→4)-α-L-fucopyranose backbone, with sulfate radicals attached at C2, C3 and C4 positions (Type II) (the figure was generated using Microsoft Office Professional 2021 and ChemDraw Pro Software).

Over 90% of the total sugar content in fucoidan consists of the deoxyhexose L-fucose [11]. Along with fucose, other monosaccharides can be found in the structure of fucoidan: galactose, glucose, xylose, mannose, rhamnose, arabinose, uronic acid and acetyl groups [12,13]. Although the structure and composition of fucoidans differ depending on the species from which they originate, fucoidans can be categorized into two main types according to their monomeric subunits (Figure 1). Type I fucoidan is composed of α-(1→3)-α-L-fucopyranose backbone linked to sulfate radicals at the C2 and C4 position, while type II fucoidan is composed of α-(1→3)- and α-(1→4)-α-L-fucopyranose backbone, with sulfate radicals attached at C2, C3 and C4 positions [12,13,14].

It was found that one species of brown algae could produce several types of fucoidan differing in their fucose, aldose, uronic acids, acetyl groups and sulfate content. Zhao et al. for example reported the extraction of four types of fucoidan from *Laminaria japonica*, which showed different antioxidant activity, depending on sulfate content and monosaccharide composition [15]. The polysaccharide sulfate content and degree of sulfation may vary among species, and it is highly dependent on the season of harvest. A study conducted by Fletcher et al. reported that the sulfate content of fucoidan from *Saccharina longicruris* increased between March and November, while a decrease was observed between November and June [16].

The molecular weights of fucoidans can range from 10 kDa to 10,000 kDa depending on the fucoidan source and extraction method. According to their molecular weight fucoidans can be categorized as: low molecular weight fucoidans (LMWF)—Mw below 10 kDa, medium molecular weight fucoidans (MMWF)—Mw between 10 and 10,000 kDa and high molecular weight fucoidans (HMWF)—Mw above 10,000 kDa [17,18].

The difference in the chemical content of fucoidans could influence their biological activity. The anticancer effect of fucoidan is strongly dependent on the polymer molecular weight [19]. Some studies report that after oral administration low-molecular-weight fucoidan is more effective compared to the HMW fractions of the polysaccharide. The reduced molecular weight facilitates the polymer intestinal absorption and the consequent expression of its activity against different cancer types, while the HMW fucoidans are effective mainly in the gastrointestinal tract [20]. According to some authors, LMW fucoidan from *Cladosiphon navaecaledoniae* induces apoptosis in breast cancer cell lines [21]. Other authors stated that treating bladder cancer cell lines with LMW fucoidan from *Sargassum hemiphyllum* inhibited angiogenesis through downregulation of the HIF-1a/VEGF signaling pathway [22]. Similar results were obtained in a study conducted on mice with induced bladder cancer treated with LMW fucoidan and chemotherapeutic agents (gemcitabine and cisplatin) [23]. According to other authors HMW fucoidan had inhibitory effect over angiogenesis while the LMW polysacharide demonstrated proangiogenic effect [24,25,26]. Furthermore, research conducted on human colon cancer cell line HCT116 using LMW fucoidan showed G1 triggered arrest and apoptosis via the p53-indipendent mechanism [27]. LMW fucoidan was also reported to induce autophagy in human stomach cancer cells (AGS cells) via microtubule-associated protein light chain 3-I transition and accumulation of beclin-1, leading to increased apoptosis [28].

Besides molecular weight, the extraction method is another essential factor which can influence fucoidan bioactivities. Generally, fucoidan is isolated through acidic hydrolysis, however this technique leads to the production of less branched fucoidan chains, which are characterized with lower bioavailability [29]. The sulfate content and the position of the sulfate groups in the polymer chain are also important for fucoidan activities [30]. For example, fucoidan extracted from Sargassum fusiforme with low sulfate content (7.5%), showed lower inhibition of HMEC-1 cells angiogenesis, compared to a polysaccharide with higher sulfate content (20.8%) [31]. The same tendency was reported for fucoidan extracted from *Fucus vesiculosus* [32]). The highly sulfated fucoidan showed higher antiproliferative effect on breast cancer cells [33]. 

The route of administration is also a basic factor, influencing fucoidan pharmacokinetics and therapeutic effects. Authors reported limited absorption of the polysaccharide and low plasma concentration after oral administration [34]. HMW fucoidan was reported to remain longer in the blood circulation, compared to LMW polysaccharide, which was quickly eliminated [35]. According to research conducted on rats, intragastric administration of fucoidan from *Fucus vesicolosus* resulted in maximum plasma concentration within four hours [36]. Tokita et al. studied the polysaccharide pharmacokinetics after a single oral administration of 1 g fucoidan by human volunteers. The results showed a maximum plasma concentration 6 or 9 h after administration [37]. Some studies have reported priority distribution of fucoidan in certain organs such as liver and kidneys [38]. There are also studies demonstrating good skin-penetrating properties of fucoidan after topical application, linear pharmacokinetics (dose applied 50–150 mg/kg) and prolonged half-life after topical delivery resulting in deposition of fucoidan in the skin 60 min after administration [39]. The type of pharmaceutical formulation could also influence the pharmacokinetics of fucoidan. According to Kimura et al., encapsulation of fucoidan in nanoparticles could elevate its cytotoxic activity and this result is related at least partially to increased permeability [40].

## 4. Fucoidan Biological Activities and Anticancer Mechanisms

**Figure 2:**
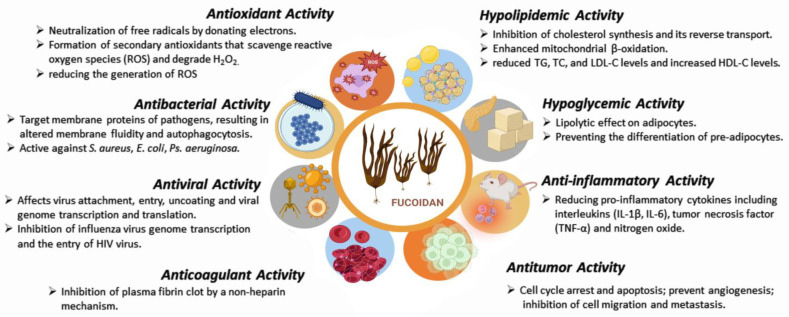
Fucoidan biological activities (created with Microsoft Office Professional 2021).

Due to its specific structure and composition, fucoidan has attracted the attention of researchers as a promising therapeutic agent and an excipient in various pharmaceutical formulations. Depending on the sulfate content and molecular weight, fucoidans show several biological effects: antioxidant [41,42,43,44], antibacterial [45,46,47,48,49,50], antiviral [51,52,53,54], anticoagulant [55,56], hypolipidemic [57], hypoglycemic [58,59], antitumor [60,61,62,63,64,65,66,67], anti-inflammatory and immunomodulatory activities [68,69,70] (Figure 2, Table 2 and Table 3). Although there are numerous publications and reports on the use of fucoidan as a therapeutic agent and carrier for drug delivery systems, no pharmaceutical dosage form containing fucoidan has been approved and released on the market so far. However, fucoidan is widely used in cosmetics, functional foods and supplements for anti-inflammatory, musculoskeletal, cardiovascular and gastrointestinal disorders. 

**Figure 3:**
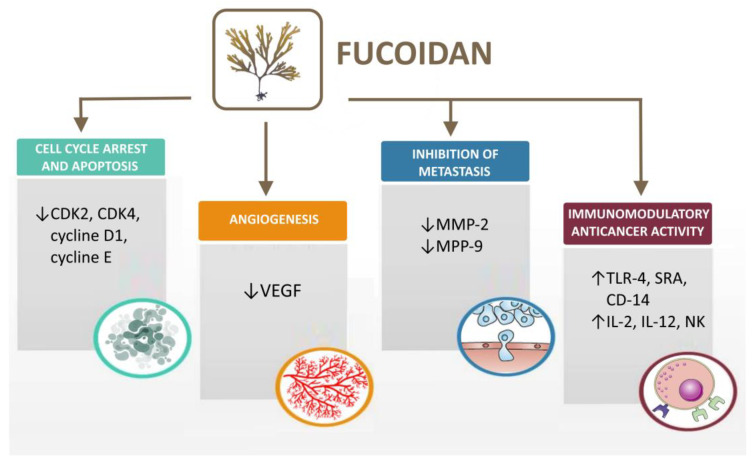
Mechanisms of fucoidan antitumor activity (created with Microsoft Office Professional 2021).

As mentioned above, fucoidan is a marine polysaccharide that is characterized by direct and indirect anticancer activity towards cancer cells (Table 4). Furthermore, the polymer possesses high biocompatibility and low toxicity which makes it a suitable adjuvant in cancer treatment [108]. Several mechanisms have been described in the literature regarding the antitumor effect of fucoidan, such as effect on the cell cycle and apoptosis [109], angiogenesis [110], cell migration and metastasis [111] and immunomodulatory anticancer activity [112] (Figure 3). 

### 4.1. Cell Cycle Arrest and Apoptosis

There are many studies on the ability of fucoidan to affect cancer cells proliferation by arresting the G1 phase of the cell cycle and reducing CDK2, CDK4 and cyclins D1 and E. Regarding the antitumor activity, one of the most thoroughly studied fucoidans are those isolated from *Fucus vesicolosus*. There have been several reports on the use of that fucoidan against different types of tumors including lymphoma, gastric, colon, liver and bladder cancers [27,113,114]. Regarding its effect on cell apoptosis, fucoidan induces programmed cell death by both intrinsic and extrinsic pathways [115]. Studies by Zue et al. and Zhang et al. showed that fucoidan promoted apoptosis in hepatocellular carcinoma and MCF-7 breast cancer in dose-dependent manner [86,116]. Furthermore, research by Li et al. revealed that not only did fucoidan not induce apoptosis in healthy cells but it also protected them by preventing programmed cell death [117].

### 4.2. Fucoidan Effects on Angiogenesis

Most tumors are characterized by uncontrolled and intensive angiogenesis and rapid cell proliferation. There are several reports regarding the capability of fucoidan to prevent angiogenesis. Moreover, fucoidan is reported to possess proangiogenic effect on tumors. Some authors state that the antiangiogenic effect is related to the decrease in the expression of endothelial growth factor (VEGF) [85], but others report that fucoidan increases the expression of VEGF [110]. A literature review published in 2014 summarized that the effect of fucoidan on tumor angiogenesis was dependent on the polysaccharide molecular weight [118]. High molecular weight fucoidans show antiangiogenic effect [119], while low molecular weight fucoidans demonstrate proangiogenic effect on tumors [110]. Furthermore, it has been demonstrated that not only the molecular weight, but also the degree of sulfation, had a significant role for fucoidan angiogenic activity. The more sulfated the fucoidans, the stronger the angiogenic effect [37,120].

### 4.3. Inhibition of Cell Migration and Metastasis

Most cancer deaths are due to metastases of cancer cells to other organs. The main mechanism of the process involves detachment of cells from the primary tumor, intravasation into blood or lymph vessels, and extravasation in other organs. Crucial to cell detachment is the process of extracellular matrix degradation, which is regulated by metalloproteinases (MPP). Fucoidan has been reported to inhibit MMP-2 and MPP-9 [121,122]. A study conducted by Cho et al. demonstrated that fucoidan inhibited metastasis in A549 human lung cancer cell line in a dose-dependent manner. Treatment of bladder cancer cells (5637) with fucoidan resulted in inhibition of cell migration by reducing MMP-9 expression [122].

### 4.4. Immunomodulatory Anticancer Activity

Studies on the immunomodulatory anticancer activity of fucoidan suggest that acetylated fucoidan induces macrophage activation through glycoprotein surface membrane receptors, such as Toll-like receptor-4 (TLR-4), scavenger receptor class A (SRA) and cluster of differentiation-14 (CD-14) [123]. Macrophage activation leads to the production of various interleukins such as IL-2 and IL-12, which further activate NK-cell-mediated apoptosis in cancer cells. A study by Zhang et al. showed that the immunomodulatory activity of fucoidan depended on the uronic acid content of the polysaccharide. Fucoidan from *Ascophyllum nodosum* with higher uronic acid content demonstrated a stronger effect on the immune system, compared to fucoidan extracted from *Fucus vesicolosus* with lower uronic acid content [123].

**Table 4:** Anticancer activity of Fucoidan from different sources.

Fucoidan Source	Dose µg/mL	Cancer Type/ Cell Line	Data Obtained	Reference
*Saccharina*	50	Colon cancer, DLD-1; Brest cancer, T-47D	Cell proliferation inhibition; Inhibition of EGF receptor binding.	[124]
*Sargassum*	200	Hepatic carcinoma, Huh6, Huh7, SK-Hep1 and HepG2	Cell proliferation inhibition via TGF-β R1, 2 ↓ Phospho-Smad2/3↓ Smad 4 protein ↓	[37]
*Sargassum*	100	Colon cancer, DLD-1	Cell proliferation inhibition; Inhibition of the formation of cytotoxic colony.	[125]
*F. vesiculosus*	20	Colon cancer, HT-29 HCT-116	Induced apoptosis via caspase-8, 9, 7, 3 activation PARP, Bak, Bid, Fas ↑, survivin, XIAP ↓.	[126]
*F. vesiculosus*	1000	Colon cancer, HT-29	Cell proliferation inhibition and induced cell apoptosis (↓Ras/Raf/ERK proteins).	[127]
*F. vesiculosus*	300	Brest cancer, MCF-7	Induced cell apoptosis via Caspase-8 activation Cytochrome C, Bax ↑ Bcl-2 ↓ Release of APAf-1 ↑.	[32]
*F. vesiculosus*	90–120	Brest cancer, MDA-MB-231	Cell proliferation inhibition via expression of phosphorylated Smad2,3 and Smad4 inhibition.	[128]
*F. vesiculosus*	50–400	Lung cancer, Lewis lung carcinoma cells	Inhibition of metastasis via inhibition of VEGF and MMPs.	[129]
*F. vesiculosus*	400	Lung cancer, A549	Cell proliferation inhibition and induced cell apoptosis via Caspase-3 ↑ and PARP cleavage.	[130]
*F. vesiculosus*	1000	Hepatic carcinoma, Huh-7, SNU-761, SNU-3085	Cell proliferation inhibition via Caspase-7, -8, -9 ↑.	[131]
*F. vesiculosus*	100–1000	Hepatic carcinoma, Huh-BAT, Huh-7, SNU-761	Induced cell apoptosis via Bax, Bid, Fas ↑ Caspase-7, -8, -9 cleavage Phosphorylatedp42/44↑.	[132]
*F. vesiculosus*	150	Leucemia, HL-60 NB4 THP-1	Induced cell apoptosis via PARP cleavage Caspase-8, 9, 3 ↑ Mcl-1, Bid ↓.	[133]
*F. vesiculosus*	50–200	Leucemia, SUDHL-4, OCI-LY8, NU-DUL-1, TMD8, U293, DB	Induced cell apoptosis via PARP cleavage and cleaved caspase-8,9, 3 ↑.	[134]
*F. vesiculosus*	12.5–100	Leucemia, NB4, HL60	Cell proliferation inhibition and induced cell apoptosis via Caspase-3, 8, 9, ↑ PARP cleavage and Bax ↑.	[135]
*F. vesiculosus*	20–100	Leucemia, U937	Cell proliferation inhibition and induced cell apoptosis via Caspase-3, 8, 9 ↑, PARP cleavage, Bax↑.	[136]
*U. pinnatifida*	200–1000	Colon cancer, WiDr LoVo Brest cancer, MCF-7	Cell proliferation inhibition.	[108]
*U. pinnatifida*	10–200	Lung cancer, A549	Cell proliferation inhibition and induced cell apoptosis via Bcl 2, p38, Phospho-PI3K/Akt, procaspase- 3↓ Bax, caspase-9, Phospho-ERK1/2 ↑ PARP cleavage.	[137]
*U. pinnatifida*	65.2–1000	Hepatic carcinoma, SMMC-7721	Cell proliferation inhibition and induced cell apoptosis via Livin, XIAP mRNA ↓ Caspase-3, -8, -9 ↑ Bax-to-Bcl-2 ratio↑ Cytochrome C ↑.	[138]
*Cladosiphon*	1000	Brest cancer, MCF-7	Induced cell apoptosis via PARP cleavage Caspase-7,8,9 ↑ Cytochrome C, Bax, Bid↑.	[139]
*B. bifurcata*	2–9	Lung cancer, NSCLC-N6	Cell proliferation inhibition via irreversible growth arrest.	[119]

↑—increased/stimulated levels; ↓—decreased/suppressed levels.

## 5. Fucoidan-Based Pharmaceutical Formulations

**Figure 4:**
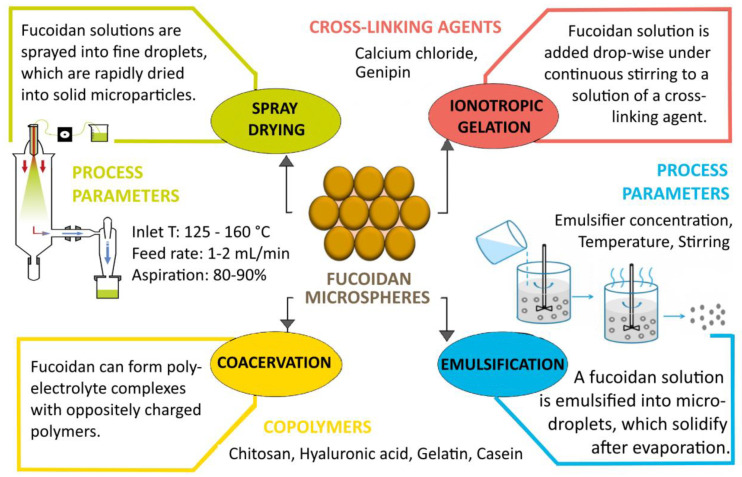
Methods for obtaining fucoidan microparticles (created with Microsoft Office Professional 2021).

In addition to its biological activity, fucoidan has suitable properties as a drug carrier, which is confirmed by numerous reports in the literature of nano- and microparticles, liposomes, films, hydrogels, etc., based on the polysaccharide (Table 5) [140,141,142,143]. The polymer backbone has anionic properties due to the presence of negatively charged sulfate ester groups. Depending on the specific chemical characteristics and the charge density of fucoidan, the polymer can interact with various biomolecules such as proteins and other polysaccharides [141]. The polysaccharide is soluble in water and acidic solutions, the solubility depending on the molecular weight and the amount of sulfate groups. Highly water-soluble fucoidans form low-viscosity solutions and are therefore generally not used as gelling agents. On the other hand, the viscoelastic properties of fucoidan are highly dependent on the origin of the polysaccharide, its concentration, molecular weight, sulfate content, pH and temperature [5]. The interaction of fucoidan with oppositely charged biopolymers such as chitosan [144], poly(2-hydroxyethyl methacrylate) [145], soybean protein isolate (SPI) [146] and lactoglobulin [147] can lead to the formation of polyelectrolyte complexes with improved characteristics for biomedical application. Polyelectrolyte complexation (complex coacervation) is one of the most widely used methods for obtaining fucoidan micro- and nanoparticles. Other common techniques for formulating particulate drug systems from the polysaccharide are simple coacervation, ionotropic gelation, spray drying, emulsification, etc. (Figure 4) [6]. Fucoidan-based micro- and nanoparticles have been used as drug delivery systems for various anticancer agents such as doxorubicin [148], methotrexate [149], curcumin [150] and cisplatin [151]. 

### 5.1. Fucoidan Microparticles 

The term microparticles refers to particles ranging in size from 1 µm to 1000 µm. Microparticles can encapsulate hydrophilic and hydrophobic agents and, depending on their structure, can be categorized as microcapsules and microspheres. Microparticles formulated from fucoidan refer to the term fucospheres. Fucospheres can be prepared using a variety of techniques and typically the manufacturing process involves the addition of copolymers or positive charge donors [5]. Such systems can provide increased stability of the drug substance, masking of its organoleptic properties, drug targeting to certain departments of the gastro-intestinal tract, controlled release and increased oral bioavailability [152]. Microspheres based on fucoidan can be used as carriers for nasal application. Combining the advantages of the microcarriers with the mucoadhesive properties of the polymer brings additional benefits such as more effective absorption and increased bioavailability due to the large total surface area of the particles, better contact with the nasal mucosa and specific targeting to the absorption site, including direct nose-to-brain drug delivery [153].

According to a study by Sezer et al., fucospheres can be prepared by crosslinking fucoidan with positively charged chitosan. The authors used bovine serum albumin (BSA) as a model drug and the resulting particles showed smooth spherical morphology and particle size ranging from 0.61 µm to 1.28 µm. Furthermore, the results showed that increasing the fucoidan content led to an increase in the zeta potential and encapsulation efficiency of BSA. The formulated particles demonstrated a sustained release of BSA characterized by a complex triphasic profile [154]. In another study, fucospheres prepared by complexation between fucoidan and chitosan were investigated for potential application in the treatment of skin burns. As previously reported, the size of the prepared microparticles increased with increasing the polymer concentration. The formulated microparticles combined the properties of the two polymers used for their preparation, resulting in a synergic effect between fucoidan and chitosan. The reported increased in vivo skin regeneration induced by the fucospheres was probably because of the effect of fucoidan on the fibroblast migration, the release of growth hormones and cytokines involved in the re-epithelization process [155]. Poly(alkyl cyanoacrylate) (PACA) microcapsules coated with fucoidan, prepared using the emulsion evaporation polymerization method, were reported to have the ability to detect thrombosis, due to the binding of fucoidan to P-selectin. The performed in vivo tests on rats demonstrated that such structures could be valuable targeting carriers for contrast agents, especially for the diagnosis of P-selectin overexpression diseases [156]. Fucospheres have also been formulated as delivery systems for antibiotics and antifungal agents. Sezer et al. used a complexation and precipitation method to prepare ofloxacin-loaded fucoidan microparticles. The formulated fucospheres showed an average particle size of 0.61 to 1.48 µm, a zeta potential of 22.3 mV and drug encapsulation efficiency of 63.9–94.8%. Regarding the drug release profile, ofloxacin-loaded fucoidan microparticles showed a sustained release, consistent with the Higuchi kinetic model [154]. Szekalska et al. used a spray-drying technique to prepare posaconazole-loaded fucoidan/gelatin microspheres for the treatment of fungal infections. The resulting particles had an average size of 12.02 µm to 17.91 µm and an encapsulation efficacy of over 90%. The microspheres were tested in simulated vaginal fluid (SVF) at pH of 4.2 and showed good swelling and mucoadhesive properties, demonstrating sustained release of posoconazole [157]. 

Since fucoidan can recognize macrophages in the alveoli, fucoidan-based microparticles may be suitable carriers for pulmonary drug delivery [158]. In a study by Cunha et al., fucoidan microparticles were proposed for the treatment of tuberculosis. Isoniazid and rifabutin were used as model drugs. The particles obtained with isoniazid had an average size of 3.78 µm, and those with rifabutin - 1.99 µm. The prepared formulations were considered as a potential pulmonary drug delivery system for the treatment of tuberculosis [159]. Fucoidan microparticles also have great potential to be used as drug delivery systems for cancer treatment. Wang et al. conducted a study on the preparation of doxorubicin loaded poly-L-ornithine/fucoidan-coated calcium carbonate microparticles using layer-by-layer self-assembly fabrication method [93]. First, calcium carbonate particles were prepared and then they were coated with poly-L-ornitine and fucoidan. The resulting blank particles were further loaded with doxorubicin, showing an encapsulation efficiency of over 70%. The in vitro study performed at pH 7.4 showed the microparticles were characterized by a prolonged release of doxorubicin..

### 5.2. Fucoidan Nanoparticles

Polymeric nanoparticles are usually spherical structures with an average size of 1 to 1000 nm. They can be categorized as nanocapsules and nanospheres depending on their composition and the location of the active pharmaceutical ingredient (API). Nanocapsules can be defined as nanosized vesicles in which the API is encapsulated in a reservoir system surrounded by a polymer membrane, while in nanospheres the drug is homogenously dissolved/dispersed in a polymer matrix. Polymer nanoparticles are multicompartment drug carriers that can provide chemical and enzymatic protection of the API and enable controlled and/or targeted drug release [5]. Due to its biocompatibility, high drug loading, stability and possibility of chemical modifications with organic and inorganic substances, fucoidan has been widely investigated for application as a drug nanocarrier [117]. In the formulation of nanoscale drug delivery systems, fucoidan is usually combined with other polymers such as poly(lactic-co-glycolic acid) (PLGA), chitosan, various proteins or liposome-forming lipids [160,161,162]. In a study by Abdelkader et al., fucoidan-based PEGylated PLGA nanoparticles for the delivery of N-methyl anthranilic acid were formulated using a single emulsion/solvent evaporation method [163]. The optimal formulation showed an average particle size of 365 nm and a zeta potential of −22.30 mV. The polymer nanosystems were characterized by a high encapsulation efficacy of 85.45% and drug loading of 51.36%. The in vitro assay showed initial burst release of 47.91% of the incorporated drug within one hour, followed by cumulative release profile for 4 h. Fucoidan-based nanoparticles containing a hydrophobic anticancer drug were developed by Lai et al. The particles were composed of fucoidan and PLGA and were designed as nanocarriers for docetaxel. The authors found that the incorporation of the drug in the nanoparticles was strongly dependent on the ratio between the two polymers used—fucoidan and poly(lactic-co-glycolic acid). The formulation prepared at a ratio of 10:3 demonstrated the highest encapsulation efficiency of 68.7 ± 4.2% [164].

The study of Huang et al. [165] gave an example of successful fucoidan-chitosan complex formation for the development of nanoparticles as carriers for oral drug delivery. The resulting polyelectrolyte complexes were formulated due to electrostatic interactions between the positively charged amino groups of chitosan and the anionic sulfate groups of fucoidan. The obtained particle models demonstrated an average particle size of 380 nm and an isoelectric point of 5.7. In another study, Huang and Kuo prepared O-carboxymethyl chitosan/fucoidan (O-CMC/F) nanoparticles for curcumin delivery [150]. Fucoidan and O-carboxymethyl chitosan were crosslinked with calcium ions to form nanoparticles with an average size between 100 and 200 nm. After loading with curcumin, the particle size increased to 270 nm. The obtained nanosystems showed high encapsulation efficacy of 92.8% and pH-dependent drug release. 

Fucoidan/trimethyl chitosan nanoparticles that enhanced transepithelial penetration of insulin by inhibiting alpha-glucosidase activity were developed by Tsai et al. [166]. The in vitro drug release results showed that insulin release was greater at neutral and intestinal pH compared to gastric fluid, where prolonged release was observed. 

Another study conducted by Chen et al. proposed the preparation of self-assembled nanoparticles from arginine-modified chitosan and thiolated fucoidan, which were designed to improve the intestinal epithelial transport of dextran and curcumin [167]. The resulting particles were characterized by pH-dependent drug release and showed improved permeation of the incorporated hydrophobic and hydrophilic drug through the Caco-2 cell monolayer barrier.

To enhance the antiangiogenic effect of fucoidan and to improve its bioavailability, Yu et al. produced oversulfated fucoidan. The modified fucoidan was further used to prepare fucoidan/chitosan nanoparticles by a polyelectrolyte complexation method [168]. The nanoparticles were stable at pH 2.5–6.0, which was probably due to the high positive charge of chitosan forming strong electrostatic interactions, while at pH 6.5 the particles were destabilized and the oversulfated fucoidan was released.

Lin et al. developed amoxicillin-loaded genipin-crosslinked fucoidan/chitosan-N-arginine nanogels for the treatment of *Helicobacter pylori* infections. Due to the strong anti-inflammatory effect and cell adhesive properties of low molecular weight fucoidans, the polymer was first depolymerized and then crosslinked with genipin to form nanogels [169]. The in vitro drug release results showed that at pH 2, an initial burst release of amoxicillin (52%) was observed, followed by a sustained release due to formation of electrostatic interactions between amoxicillin and low molecular weight fucoidan. At pH 6.5, the nanogels were stable, allowing only a small amount of amoxicillin to be released, while at pH 7.4 the nanogels disassembled and rapidly released the incorporated drug. 

Eggshell protein, which has properties enabling it to treat inflammatory bowel disease, undergoes rapid enzymatic degradation when taken orally, severely limiting its bioavailability. A study conducted by Lee et al. demonstrated that fucoidan–chitosan nanoparticles could be used as carriers for that protein. Polymer nanostructures were characterized by a pH-dependent in vitro drug release such that at pH 1.2–2.5 no release was observed, while at pH 7.4 50% of the protein was released in a sustained manner [170].

Fucoidan can also form polyelectrolyte complexes with proteins [146,171]. A study conducted by Fan et al., for example, proposed a formulation of fucoidan/soybean nanoparticles as a delivery system for curcumin [146]. The average size of the obtained particles ranged from 200 to 400 nm and the encapsulation efficiency was up to 97.6%. The in vitro release assay performed in simulated gastric fluid showed that the nano formulations released more than 80% of the incorporated curcumin within 4 h. A storage stability test demonstrated that after one week soybean nanoparticles showed physical instability, while the fucoidan–protein structures remained stable, which confirmed the stabilizing function of fucoidan in the nanoparticle complex. 

### 5.3. Fucoidan Liposomes

Liposomes are other commonly used nanosized drug delivery systems that can offer advantages such as improved solubility, increased drug stability and targeted delivery [172]. A study by Qadir et al. demonstrated that, when encapsulated in liposomes, fucoidan from *F. vesiculosus* had stronger anticancer activity, reducing interleukin-6 and tumor necrosis factor-α levels, compared to fucoidan nanoparticles [173]. Salviano et al. developed a liposomal drug delivery system composed of fucoidan derivative (cholesteryl-fucoidan) loaded with usnic acid for the treatment of *Mycobacterium tuberculosis* infection. The formulated systems showed significantly lower IC50 (8.26 ± 1.11 µM) compared to blank liposomes, due to higher uptake and cellular internalization [174]. In another study, Zhang et al. formulated liposomes as delivery systems for bioactive nutraceuticals by coating them with fucoidan. The authors highlighted the stabilizing effect of fucoidan, which can reduce the drug leakage from the liposomes and the initial burst release, thereby improving the stability and bioavailability of liposomes [175].

**Table 5:** Fucoidan micro and nano-sized formulations and their biomedical applications.

Active Substance	Dosage Form	Therapeutic Application	Data Obtained	Reference
Fucoidan	Microparticles	Skin burns	Enhanced skin regeneration resulted in an increase in the epithelial thickness in vivo on rabbits.	[155]
Perfluorooctyl bromide	Microparticles	Thrombosis	High affinity to P-selectin and ability to detect thrombosis in vivo on rats with induced aortic aneurysm.	[156]
Isoniazid, Rifabutin	Microparticles	Tuberculosis	Effective inhibition of mycobacterial growth. Low toxicity on human alveolar epithelum and monocytic cell line (HTP-1).	[159]
Doxorubicin	Microparticles	Breast cancer	Significant antiproliferative activity on MCF-7 breast cancer cell line. Low toxicity on mouse myoblasts (concentration 400 µg/mL).	[162]
N-methyl anthranilic acid	Nanoparticles	Inflammation	Significant reduction in carrageenan-induced inflammation in rats by decreasing the cyclooxygenase-2, (TNF)-α E2, NO, IL-1β, IL-6.	[163]
Docetaxel	Nanoparticles	Anticancer	High cellular uptake efficiency and subcellular localization. Low cytotoxicity on non-tumor cell lines.	[164]
Oversulfated fucoidan	Nanoparticles	Anticancer	Inhibition of human umbilical vein endothelial cells HUVECs via competitive binding to bFGF receptors (bFGFRs).	[168]
Amoxicillin	Nanogels	*H. pylori* infection	pH-dependent sustained drug release, which can improve the therapy of *Helicobacter pylori*.	[169]
Piperlongumine	Nanoparticles	Prostate cancer	Higher cytotoxicity in the PC-3 cells than in the non-cancerous hDFB cells; increased the intracellular ROS levels in the PC-3 cells.	[176]
Usnic acid	Liposomes	*M. tuberculosis*	Higher cellular uptake and cellular internalization.	[174]

## 6. Fucoidan Nanoparticles for Cancer Therapy

**Figure 5:**
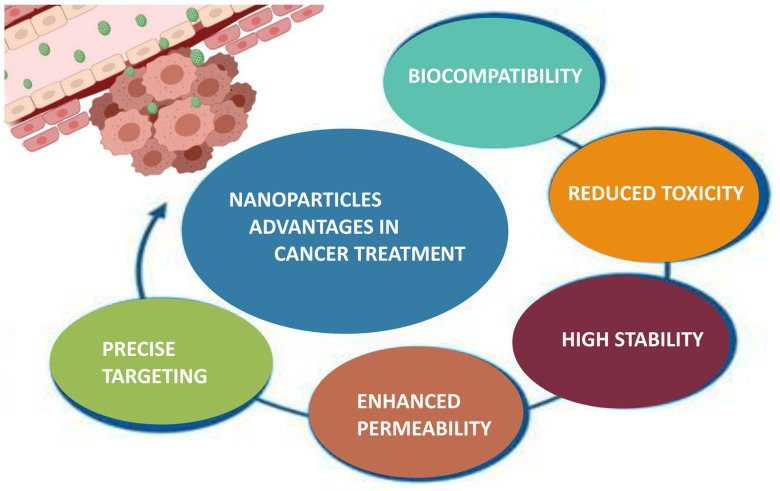
Advantages of nanoparticles in cancer treatment (created with Microsoft Office Professional 2021).

Fucoidan nanoparticles have been extensively studied as drug delivery systems for cancer treatment, due to their multifaceted advantages such as controlled drug release, improved drug permeability and precise targeting to specific cancer cells (Figure 5) [177]. Fucoidan has been outlined as a suitable carrier for various anticancer agents, including doxorubicin (DOX), copper sulfide (CuS), methotrexate (MTX), curcumin (CUR), cisplatin, etc. (Table 6). 

Kim et al. developed fucoidan-coated gold nanoparticles loaded with doxorubicin for photothermal therapy of squamous carcinoma [148]. In vitro cytotoxic assay was conducted on RAW 264.7 macrophage cells and on VX2 squamous cell line. As expected, as the concentration of the nanoparticles increased, cell viability decreased. Furthermore, after laser irradiation of the VX2 cells containing fucoidan-coated gold nanoparticles, maximal cell death was recorded (88%).

Fucoidan nanoparticles have also been used as carriers for cisplatin [151]. The prepared nanoparticles showed a high encapsulation efficacy of 93.3%, an average size of 181.2 nm and a zeta potential of −67.4 mV. An immune protection assay was performed on RAW 264.7 macrophage cells to investigate the cytotoxic effect of encapsulated cisplatin. HCT-8 tumor cells were treated with different concentrations of cisplatin, fucoidan and cisplatin-loaded fucoidan nanoparticles. The formulated nanoparticles caused greater toxicity to the tested cancer cells compared to the free drug. 

Using the polyelectrolyte complexation method, Lue et al. developed multi-stimuli-responsive fucoidan/protamine nanoparticles as carriers for doxorubicin [178]. According to TEM and DLS analyses, the average particle size was 180 nm and the zeta potential ranged from −22 mV to −43 mV depending on the fucoidan/protamine ratio. The cytotoxic effect of the nanoparticles was evaluated using MDA-MB-231 cell line. Enzymatic digestion and the acidic intracellular microenvironment of the cancer cell triggered the release of doxorubicin from the nanoparticles, resulting in decreased cell viability. P-selectin expression was determined using a mouse monoclonal antibody (anti-selectin/CD62P) against MDA-MB-231 cells and the result showed that doxorubicin-loaded fucoidan/protamine nanoparticles inhibited platelet activation. The authors suggested that the proposed nanostructures can be used as potential drug delivery systems for intracellular release of anticancer drugs against metastatic breast carcinoma. 

In order to develop an immunomodulatory platform for the delivery of doxorubicin, Pawar et al. formulated fucoidan-polyethileneimine nanoparticles using a polyelectrolyte complexation technique [179]. The in vitro release study showed that the drug was released faster at acidic pH 5.5, compared to neutral pH 7.4. In both media the particles demonstrated an initial burst release within the first 24 h, followed by a sustained release. Furthermore, doxorubicin-loaded nanoparticles showed a greater cytotoxic effect on MDA MB-231 and 4T1 cell lines compared to free daunorubicin. The in vivo anticancer assay performed on 4T1 induced tumor in BALB/c mice confirmed the targeted delivery of doxorubicin to the tumor site.

Self-assembled nanoparticles based on a combination of fucoidan with polyallylamine were formulated as drug carriers for methotrexate. The developed polymer nanosystems demonstrated significant inhibition of the proliferation of HeLa and MCF-7 cell lines [149]. In another study, fucoidan was used to functionalize doxorubicin-loaded micelles [184]. The average size of the developed structures was 120 nm and their zeta potential was −20 mV. The biodistribution of the obtained micelles and free doxorubicin was studied in tumor-induced BALB/c mice 1, 8 and 24 days after administration. Fucoidan-functionalized doxorubicin micelles showed high accumulation at the tumor site and limited distribution in the kidneys and liver, making them a promising strategy for the treatment of metastatic cancer.

Kang et al. developed doxorubicin-loaded fucoidan-coated nanoparticles to overcome multidrug resistance in chemotherapy [185]. The obtained particles had an average size of 35 nm and a drug encapsulation efficiency of 35%. The cytotoxicity assay showed that the particles reduced the viability of the human breast cancer cell line (MCF-7ADR), while only mild cytotoxicity was observed in the normal breast cell line (MCFA-10). An in vivo study on BALB/c male mice with induced tumor (transplanted MCF-7ADR cells in the right flank) demonstrated efficient delivery of doxorubicin to the targeted tumor tissues and limited accumulation in the liver.

One of the key factors in cancer growth and metastasis is the P-selectin protein which has a significant role in the activation of platelets associated with cancer cell metastasis. Therefore, targeting and inhibiting the action of P-selectin protein is an essential mechanism for achieving effective cancer therapy. The ability of fucoidan to target P-selectin proteins was among the main reasons for Jafari et al. to use it as a nanocarrier of doxorubicin for the treatment of breast cancer [186]. The formulated polymer particles had an average size of 167.3 nm, a drug loading of 4.81% and a zeta potential of −19.87 mV. By adding polyethylene glycol (PEG), the zeta potential increased to −15.09 mV, indicating that PEG partially shielded the particle surface charge. In vitro drug release was carried out in PBS of pH 7.4 and pH 5.2. No initial burst release was observed in both media in the first hours, probably due to the covalent bonds between doxorubicin and fucoidan. After 72 h, 35% of doxorubicin was released in PBS 7.4, while in PBS 5.2 the release was faster (60%), demonstrating a pH-dependent pattern. The P-selectin expression was studied via P-selectin recombinant rabbit monoclonal antibody using two breast cancer cell lines (MDA-MB-231, MDA-MB-468), compared with normal breast cells (MCF-12A). Western blotting and immunofluorescent imaging revealed that P-selectin was better expressed on MDA-MB-231 cells, compared to the other two cell lines. The developed fucoidan nanoparticles demonstrated high cellular drug uptake and can be used for targeted and sustained release of doxorubicin.

DuRoss et al. investigated the P-selectin targeting properties of temozolomide/talazoparib-loaded fucoidan-coated 1,4-dicarboxybenze metal-organic nanoparticles to achieve enhanced chemotherapeutic effect against colorectal cancer [187]. The obtained particles had a negative zeta potential, a particle size of 30–70 nm and an encapsulation efficiency of 90% for temozolomide and 54% for talazoparib. According to the in vitro test performed in PBS pH 7.4, the formulated nanoparticles released 40% of the incorporated drugs within the first 24 h, followed by sustained release. After 7 days talazoparib was completely released from the particles in contrast to temazolomide (only 60%). In vitro cytotoxicity test was conducted on CT26 cell line, and P-selectin expression was evaluated on bEnd.3 and CT26 cancer cell lines. After treatment of the cells with the developed nanoparticles and subsequent administration of increasing doses of radiation (0, 1, 2, 3, 4, 5 Gy), a significant decrease in cell survival was registered. In vivo biodistribution analysis in tumor-bearing mice showed that the administered nanoparticles had a high accumulation at the tumor site both with or without irradiation, offering improved drug delivery and enhanced radiotherapy.

To achieve improved drug distribution and pharmacokinetics, Chen et al. proposed encapsulation of epigallocatechin-3-gallate (EGCG) and docetaxel (DTX) in D-Alpha-tocopheryl polyethylene glycol 1000 succinate-conjugated fucoidan/hyaluronic acid nanoparticles for the treatment of prostate cancer [188]. The particle size ranged from 189.96 nm to 238.98 nm and the zeta potential from −25.22 mV to −27.42 mV. EGCG:DTX concentration ratio of 2.0:0.2 mg/mL was determined as optimal, resulting in encapsulation efficiency of 43.39% for EGCG and 85.86% for DTX. The formulated nanoparticles were characterized with pH-dependent in vitro drug release. In 24 h at pH 5.5, they released 37.33% EGCG and 43.75% DTX. The obtained particles demonstrated internalization in prostate cancer cells by recognizing CD44 and P-selectin ligand. Furthermore, the in vitro test indicated that the polymer particles inhibited cell growth by inducing G2/M phase arrest of the cells. Additional in vivo studies confirmed that the formulated drug-loaded nanostructures significantly reduced tumor growth and increased M30 protein expression.

Oliveira et al. formulated antibody (ErbB-2) conjugated fucoidan–chitosan nanoparticles loaded with gemcitabine for the treatment of metastatic cancer [189]. The nanoparticle had an average size of 160 nm, a polydispersity index of 0.18 and a positive zeta potential of 21 mV. The targeting capability of the nanoparticles was confirmed by an increased cellular uptake of ErbB-2 positive SKBR3 breast cancer cells. The cytotoxicity assay indicated that the Ab-conjugated nanoparticles exerted increased cytotoxic effect in breast cancer cells (above 80%) compared to free gemcitabine (only 15%) and nanoparticles blended with gemcitabine (20%). The in vivo study on immunocompromised female mice with induced breast cancer demonstrated that the targeting drug delivery system significantly reduced tumor growth and lung metastasis. 

Cavalcanti et al. developed fucoidan-coated poly(isobutyl cyanoacrylate) nanoparticles loaded with oncocalyxone A for the treatment of metastatic breast cancer [190]. The produced nanoparticles had an average size of 305 ± 6.49 nm and encapsulation efficiency of 100%. The authors performed a cytotoxicity assay on J774A.1 macrophage cells and MDA-MB-231 breast cancer cell line. The results showed that neither free oncocalyxone A nor oncocalyxone A-loaded nanoparticles had cytotoxic effect on J774A.1 macrophage cell line. The results from the MTT test indicated that the oncocalyxone A-loaded fucoidan nanoparticles exerted higher cytotoxic activity against MDA-MB-231 cells after 72 h of exposure compared to pure oncocalyxone A.

Other anticancer agents—paclitaxel and mitogen-activated protein kinase—were also encapsulated in fucoidan through a nano-precipitation technique [191]. To study the targeting efficacy of the obtained nanoparticles SK-136 murine cell line was used. The results showed that fucoidan-based nanoparticles can specifically target the tumor microenvironment and be localized via MEK inhibitors in both primary and metastatic cancer cells, demonstrating improved anticancer activity.

The provided examples of successful incorporation of anticancer agents into fucoidan nanoparticles convincingly demonstrate the potential of this polysaccharide as a drug carrier. However, most of the reported formulations have been analyzed mainly by in vitro assays and to a lesser extent characterized in vivo. Further research and clinical trials are needed to prove the effectiveness and safety of fucoidan nano- and micro-particles as therapeutic systems and to enable their utilization on the pharmaceutical market as final pharmaceutical products. Although such studies are still scarce in the literature, the positive data from clinical trials on the antitumor effectiveness of fucoidan suggest that fucoidan formulations have great potential as a safe and effective drug-delivering system for application in oncology (Table 7).

## 7. Conclusions

Most conventional cancer therapies are associated with low specificity, which can lead to significant side effects and toxicity. Micro- and nanotechnologies allow the development of new formulations that permit slow and selective release of various drugs at specific tumor sites. Recently, marine natural products have been intensively investigated for their antitumor activity and optimum properties as drug carriers for the development of innovative anti-cancer medicines. In vivo studies have demonstrated the anticancer effect of fucoidan, which may be due to the inhibition of tumorigenesis and metastasis, and suggest that the polysaccharide is a potential preventive or therapeutic agent in oncology. Although further research is needed to fully elucidate the bioactivities, pharmacokinetics, safety and dosage of fucoidan, it can certainly be concluded that due to its unique characteristics as a drug carrier and its antineoplastic properties, this polysaccharide is believed to have a promising future in cancer treatment.

## Figures and Tables

**Table 1:** Structural characteristics of fucoidan extracted from different types of brown seaweeds.

Brown Seaweed	Structure	Reference
*Fucus evanescens*	Main chain of (1→3)-and (1→4)-α-L-Fucp highly substituted by sulfate groups at O2 and/or O3 positions	[6]
*Fucus seratus*	Main chain of (1→3)-and (1→4)-α-L-Fucp with short branches of α-L-Fucp-(1→4)-α-L-Fucp and α-L-Fucp-(1→3)-α-L-Fucp in O4 of α-(1→3)-L-Fucp and sulfate groups in O2 and/or O4 positions	[7]
*Chorda filum*	Main chain of (1→3)-α-L-Fucp highly ramified at O2 by terminal residues and substituted by sulfate groups at O2 and/or O4 positions.	[7]
*Fucus distichus*	[(1→3)-α-L-Fucp-(2,4-di-SO_3_-)-(1→4)- α-L-Fucp -(2SO_3_-)-(1→]n	[8]
*Undaria pinnatifida*	Backbone structure of (1→3):(1→4)-O-glycosidic bonds	[9]
*Ascophyllum nodosum*	[(1→3)-α-L-Fuc-(2SO_3_-)-(1→4)- α-L-Fuc-(2,3-di-SO_3_-)-(1→]n	[10]
*Laminaria saccharina*	Main chain of (1→3)-α-L-Fucp branched at O2 and O4 of α-L-Fucp by terminal residues and sulfate groups	[11]

**Table 2:** In vitro biological activities of fucoidan.

Activity	Dose, µg/mL	Source	Cell Lines	Data Obtained	Reference
Antioxidant	1	*S. japonica*	-	DPPH radical and ABTS+ radical scavenging activity	[71]
Anti- inflammatory	0.1–100	*S. sagamianum*	LPS-induced RAW 264.7 cells	Inhibition of NO, IL-6, IL-1β, TNF-α, iNOS, COX-2, NF-κB p65	[72]
Anti- inflammatory	1–300	*G. pacificum*	LPS-induced THP-1 cells	Protected the THP-1 cells against LPS-stimulated cytotoxicity	[73]
Anti- inflammatory	3–25	*S. japonica*	LPS-induced RAW 264.7 cells	Decreased production of NO and TNF-α, IL-1β and IL-6.	[74]
Anti- inflammatory	25–100	*C. minima*	LPS-induced RAW 264.7 cells	Inhibition of NO production and expression of PGE2	[75]
Antiviral	10–1000	*L. japonica*	Vero E6 cell line from mouse macrophage	Reduced number of infected cells	[76]
Antiviral	50–100	*H. elongata*	Vero cell line from African green monkey	Inhibition of Herpes simplex virus type 1 intracellular replication	[77]
Hypoglycemic	0.5–5	*L. japonica*	Cell α-glucosidase	α-glucosidase inhibitory activity	[78]
Immuno- modulatory	5–125	*S. fusiforme*	B lymphocytes	Promote LPS-induced spleen lymphocyte proliferation	[79]
Anticancer	25–100	*S. coreanum*	HL-60, CT-26, B-16 and HeLa cell model	Cell apoptosis and activation of caspase-3 and cleavage of poly(ADP-ribose) polymerase	[80]
Anticancer	120	*F. vesiculosus*	HeLa G-63, Hep G2 and Chang liver cells model	Inhibition of cell proliferation	[81]
Anticancer	10–500	*U. pinnatifida*	Sarcomas and carcinosarcoma cell lines	Induced apoptosis	[82]
Anticancer	0.05–100	*B. fuscopurpurea*	Ovarian cancer cells	Inhibition of A2780, COC1, SKOV3, HO-8910 and OVCAR3 ovarian cancer cells proliferation	[83]

**Table 3:** In vivo tests, performed with fucoidan from different sources.

Effect	Dose	Source	Tested Model	Reported Results	Reference
Anticancer	5 g/kg p.o.	*C. okamuranus*	Mice with induced colon cancer	Significantly suppressed tumor growth	[84]
Anticancer	5 mg/kg i.p.	*F. vesicolosus*	Mice with induced breast cancer	Inhibition of angiogenesis and induction of apoptosis	[85]
Anticancer	200 mg/kg i.p.	*-*	Mice with induced hepatocellular carcinoma	Inhibition of cancer cells proliferation	[86]
Anticancer	0.25 mg/day i.v.	*F. vesicolosus*	Mice with induced breast cancer	Cancer metastasis prevention	[87]
Anticancer	75 mg/kg p.o.	*S. plagiophyllum*	Mice with induced hepatocellular carcinoma	Inhibition of carcinogen metabolism	[88]
Anticancer	100 mg/kg p.o.	*C. okamuranus*	Mice with induced sarcoma	Tumor growth reduction by NO produced by macrophages	[89]
Immuno- modulatory	10 mg/kg i.v.	-	Rabbits with induced bacterial meningitis	Decreased influx of leukocytes into the cerebrospinal fluid	[90]
Immuno- modulatory	50 mg/kg i.v.	-	Mice with L-selectin deficient	Mobilization of hematopoietic progenitor stem cells	[91]
Immuno- modulatory	25 mg/kg i.v.	*F. vesiculosus*	Rats with induced myocarditis	Inhibition of extravasation of macrophages and CD4+ T cells	[92]
Immuno- modulatory	200 mg/kg p.o.	-	Mice with induced leishmania infection	Th1 switch in Leishmania infection	[93]
Immuno- modulatory	20 mg/kg i.p.	*F. vesiculosus*	Mice	Increased levels of TNF-α and IL-6 in spleens and blood serum	[94]
Immuno- modulatory	20 mg/kg p.o.	*U. pinnatifida*	Mice with induced herpes simplex	Increased activity of NK cells	[95]
Immuno- modulatory	50 mg/kg i.p.	*M. pyrifera*	Mice	Increased maturation and activation of NK cells	[96]
Immuno- modulatory	500 mg/kg p.o.	*U. pinnatifida*	UVB-irradiated mouse skin	Lowered IFN-γ levels after irradiation; reduced skin edema	[97]
Anti- inflammantory	25 mg/kg i.v.	*F. vesiculosus*	Mice with induced pancreatitis	Decreased levels of IL-1β, TNF-α and myeloperoxidase	[98]
Anti- inflammantory	10–400 mg/kg p.o./i.p.	*F. vesiculosus*	Mice with induced acute colitis	Decreased levels of IL-1α, IL-1β and IL-10	[99]
Anti- inflammantory	50–100 mg/kg p.o.	*L. japonica*	Mice with induced liver damage	Reduced levels of TNF-α, IL-1β and IL-6	[100]
Anti- inflammantory	100 mg/kg p.o.	*L. japonica*	Mice with induced diabetes melitus	Reduced blood glucose level and serum levels of IL-1β, IL-6, TNF-α	[101]
Anti- inflammantory	50–200 mg/kg p.o.	*L. japonica*	Mice with induced myocarditis	Decreased levels of TNF- α and IL-6; increased levels of IL-10	[102]
Anti- inflammantory	50–200 mg/kg p.o.	*S. muticum*	Mice with induced rheumatoid arthritis	Decreased levels of TNF-α, IFN-γ and IL-6	[103]
Anti- inflammantory	-	*C. okamuranus*	Mice with induced chronic colitis	Decreased levels of IL-6 and increased levels of IL-10	[75]
Anti- inflammantory	12.5–50 μg/mL	*C. minima*	Zebrafish embryos	Decreased production of NO, ROS, COX-2, iNOS	[104]
Anti- inflammantory	300 mg/mL p.o.	*U. pinnatifida*	Mice with induced rheumatoid arthritis	Reduced cartilage and bone destruction and inflammation	[105]
Anti- inflammantory	2.5–300 mg/kg p.o.	*T. ornata*	Rats with induced arthritis	Decreased levels of TNF-α, IL-6 and PGE2	[106]
Anti- inflammantory	50 mg/kg p.o.	*T. ornata*	Mice with paw edema	Reduced the expression of genes of COX-2, IL-1β, the NF-κB signaling pathway	[107]

**Table 6:** Fucoidan-based nanoparticulate drug delivery systems for cancer therapy.

Source, Mw	Copolymer	Drug	Preparation Method	Administration Route	Reference
*F. vesiculosus*, 200–400 kDa	Gold nanoparticles	Doxorubicin	Electrostatic complexation	Ocular	[148]
*L. japonica*, 80 kDa	Protamine	Doxorubicin	Self-assembly	Intravenous	[178]
*F. vesiculosus*, 200–400 kDa	Polyethyleneimine	Doxorubicin	Coacervation	Intravenous	[179]
*F. vesiculosus*, 200–400 kDa	Polyallylamine HCl	Copper sulfide	Layer-by-layer	Intratumoral	[180]
*F. vesiculosus*, 200–400 kDa	Polyallylamine HCl	Methotrexate	Self-assembly	NA *	[149]
*F. vesiculosus*, 200–400 kDa	Chitosan/Chondroitin	Fucoidan	Coacervation	Oral	[181]
*F. vesiculosus*, 50–190 kDa	Chitosan	Methotrexate	Self-assembly	Topical	[182]
*F. vesiculosus*, 50–190 kDa	Chitosan	Curcumin	Self-assembly	Oral	[183]

* NA—no available information.

**Table 7:** Clinical trials/Case reports on fucoidan anticancer activity.

Fucoidan Source	Cancer Type	Dose Applied	Study Type	Number of Patients	Clinical Results	Reference
LMW fucoidan from *S. hemiphyllum*	Metastatic colorectal cancer	4 g/day	Randomized, double-blind, controlled trial	54	Improved disease control	[192]
LMW fucoidan from *C. okamuranus*	Advanced metastatic cancer	400 mL/day	Open-label clinical study	20	Improved therapy	[67]
LMW fucoidan from *C. okamuranus*	NK cells from patients in remission	1.5 g twice a day	Randomized double-blind placebo-controlled study	39	Enhanced NK cell activity	[193]
HMW fucoidan from *C. okamuranus*	Colorectal cancer	150 mL/day	A randomized trial	20	Decreased general fatigue	[194]
Fucoidan (*without clarification*).	Rectal cancer	N/A	A double-blind randomized placebo-controlled study	100	N/A	[195]
Fucoidan as a dietary supplement	Squamous cell carcinoma	4.4 g/day	A randomized double-blind study	119	Study not completed.	[196]
Fucoidan as a dietary supplement	Hepatocellular carcinoma	4.4 g/day	A randomized double-blind study	100	Study not completed.	[197]
LMW fucoidan as a dietary supplement	Lung cancer	4.4 g twice a day	A double-blind randomized controlled trial	N/A	Withdrawn.	[198]
LMW fucoidan from *Undaria pinnatifida*	Brest cancer	500 mg twice a day	Open label non-crossover study	20	Co-administration with letrozole, tamoxifen.	[199]
LMW fucoidan from *Nemacystis decipiens*	Prostate, liver, breast, pancreatic cancer	60–300 mL/day	Case report	10	Decreased tumor markers	[42]

LMW—low molecular weight; HMW—high molecular weight; N/A—not available information.

## Data Availability

All data are in the main body of the paper.
